# LoopGrafter: a web tool for transplanting dynamical loops for protein engineering

**DOI:** 10.1093/nar/gkac249

**Published:** 2022-04-19

**Authors:** Joan Planas-Iglesias, Filip Opaleny, Pavol Ulbrich, Jan Stourac, Zainab Sanusi, Gaspar P Pinto, Andrea Schenkmayerova, Jan Byska, Jiri Damborsky, Barbora Kozlikova, David Bednar

**Affiliations:** Loschmidt Laboratories, Department of Experimental Biology and RECETOX, Faculty of Science, Masaryk University, 625 00 Brno, Czech Republic; International Clinical Research Center, St Anne’s University Hospital Brno, 656 916 Brno, Czech Republic; Department of Visual Computing, Faculty of Informatics, Masaryk University, 602 00 Brno, Czech Republic; Department of Visual Computing, Faculty of Informatics, Masaryk University, 602 00 Brno, Czech Republic; Loschmidt Laboratories, Department of Experimental Biology and RECETOX, Faculty of Science, Masaryk University, 625 00 Brno, Czech Republic; International Clinical Research Center, St Anne’s University Hospital Brno, 656 916 Brno, Czech Republic; Loschmidt Laboratories, Department of Experimental Biology and RECETOX, Faculty of Science, Masaryk University, 625 00 Brno, Czech Republic; Loschmidt Laboratories, Department of Experimental Biology and RECETOX, Faculty of Science, Masaryk University, 625 00 Brno, Czech Republic; International Clinical Research Center, St Anne’s University Hospital Brno, 656 916 Brno, Czech Republic; Loschmidt Laboratories, Department of Experimental Biology and RECETOX, Faculty of Science, Masaryk University, 625 00 Brno, Czech Republic; International Clinical Research Center, St Anne’s University Hospital Brno, 656 916 Brno, Czech Republic; Department of Visual Computing, Faculty of Informatics, Masaryk University, 602 00 Brno, Czech Republic; Loschmidt Laboratories, Department of Experimental Biology and RECETOX, Faculty of Science, Masaryk University, 625 00 Brno, Czech Republic; International Clinical Research Center, St Anne’s University Hospital Brno, 656 916 Brno, Czech Republic; Department of Visual Computing, Faculty of Informatics, Masaryk University, 602 00 Brno, Czech Republic; Loschmidt Laboratories, Department of Experimental Biology and RECETOX, Faculty of Science, Masaryk University, 625 00 Brno, Czech Republic; International Clinical Research Center, St Anne’s University Hospital Brno, 656 916 Brno, Czech Republic

## Abstract

The transplantation of loops between structurally related proteins is a compelling method to improve the activity, specificity and stability of enzymes. However, despite the interest of loop regions in protein engineering, the available methods of loop-based rational protein design are scarce. One particular difficulty related to loop engineering is the unique dynamism that enables them to exert allosteric control over the catalytic function of enzymes. Thus, when engaging in a transplantation effort, such dynamics in the context of protein structure need consideration. A second practical challenge is identifying successful excision points for the transplantation or grafting. Here, we present LoopGrafter (https://loschmidt.chemi.muni.cz/loopgrafter/), a web server that specifically guides in the loop grafting process between structurally related proteins. The server provides a step-by-step interactive procedure in which the user can successively identify loops in the two input proteins, calculate their geometries, assess their similarities and dynamics, and select a number of loops to be transplanted. All possible different chimeric proteins derived from any existing recombination point are calculated, and 3D models for each of them are constructed and energetically evaluated. The obtained results can be interactively visualized in a user-friendly graphical interface and downloaded for detailed structural analyses.

## Graphical Abstract

**Graphical Abstract:**
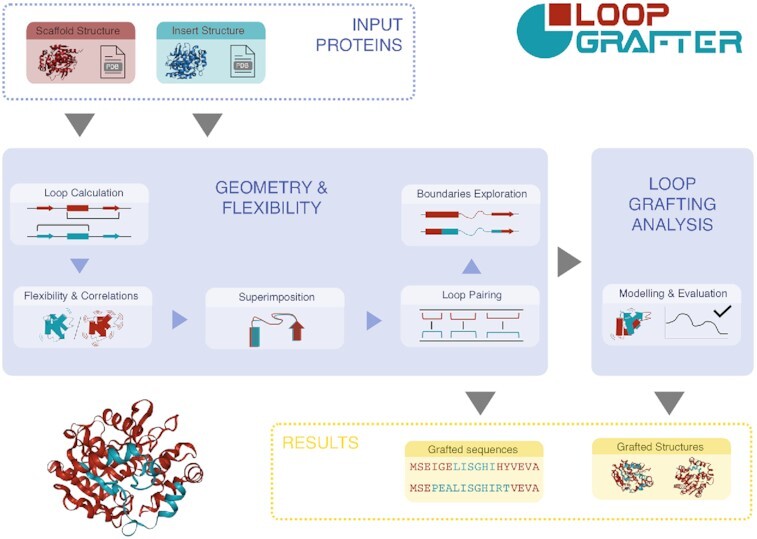
LoopGrafter calculates loop properties on homologous input structures informing the choice of regions to transplant. Exhaustively exploring the recombination space and evaluating 3D structures facilitate selecting successful protein chimeric designs.

## INTRODUCTION

Enzymes are powerful natural biocatalysts that have evolved through billions of years to perform the complex chemical reactions required to sustain life. Improving different properties of such biocatalysts has been one of the main objectives of protein engineering, and both directed evolution (1) and computationally aided approaches (2) have helped in this aim. Loops, particularly dynamic aperiodic regions connecting more steady regular secondary structures, have been repeatedly targeted in protein engineering (3). Yet, the scarcity of available methods for loop-based rational protein engineering has been noted (4).

The transplantation or grafting of such elements from a source protein to a target one has long been considered a powerful approach to engineer the activity and specificity of enzymes (5–7). Based on the study of successful loop transplants, it has previously been proposed that loop grafting requires a precise local geometric overlay of the source and target structures around the grafted region (8). This suggests that preserving the geometry and local conformation of the secondary structure elements (SSEs) flanking the grafted loop is crucial for the grafting success. Such conformational and geometric requirements were observed in the early *de novo* loop design, where the extent of backbone constraints was arbitrarily set to the first three proximal amino acids of each flanking secondary structure (9). However, individual grafting experiments differ on the extent of this requirement, making it unclear how much of the flanking secondary structure needs to be grafted along the coil part of the fragment. Additionally, we have recently shown that flexibility requirements in the context of loop grafting may be crucial for tailoring the desired enzyme function (10,11). Indeed, protein flexibility is considered to play an important role in the evolution of proteins towards new functions, in particular the flexibility gradient between the active site and the protein scaffold (12).

In this context, the aforementioned geometric requirements can only help in keeping backbone conformations suitable for the preservation of an adequate balance between stability and function (13). Furthermore, it has been shown that allosteric coordination of distant parts of a protein modulates the dynamic conformational space of loops important for the enzyme function (14–16). In the context of loop grafting, the identification and potential design of such regions might be crucial for achieving the desired engineered function. There are promising results of loop grafting efforts, and several solutions are available for loop modelling and remodelling, such as Loop Modelling (17), DaReUS-Loop (18) or FALC (19). Nevertheless, no computational tool is at the disposal of the community to provide guidance on the grafting process while assessing the flexibility of the grafted regions.

Here, we introduce LoopGrafter (https://loschmidt.chemi.muni.cz/loopgrafter/), a web-based tool aimed to cover this gap in a protein engineering toolbox. Specifically, the web server automates the identification of regular SSEs in the input protein structure, allowing the user to fine-tune the limits of such elements. Loops and their geometries are calculated to provide the user with an easy way to compare the elements on the scaffold and insert proteins to be exchanged. LoopGrafter is designed to help researchers to identify regions of interest in their scaffold protein according to their dynamic behaviour: rigid, flexible or hinge regions connecting parts of the protein with different flexibility properties (i.e. one part rigid, the other flexible). Furthermore, motion cross-correlations between the selected loops to be grafted and other loops in the protein are assessed to suggest additional regions to be engineered. LoopGrafter calculates all possible combinations of the selected loops and, for each loop, every recombination point on the two flanking SSEs that generate a different grafted protein sequence. This exhaustive exploration of the recombination sequence space allows LoopGrafter not to require the sequence of the final chimeric protein as input, as it happens in available loop remodelling tools that can be used for loop grafting (17–19). Three-dimensional (3D) models for each of such grafting solutions are generated and energetically evaluated to indicate and rank the experimental feasibility of the designed sequence.

## MATERIALS AND METHODS

### Determination of SSEs and flexibility assessment

The 3D structures of the two input proteins can be uploaded from the Protein Data Bank (PDB) (20) or uploaded by the user. The secondary structure of input protein structures is calculated using DSSP (21,22). As in previous works (23), in our pipeline a loop is defined as the super-secondary structure formed by two regular secondary structures and the coil region joining them. Based on these definitions, the input protein loop architecture and the geometries of their loops are calculated as described in the ArchDB structural classification of loops in proteins (23). To assess the scaffold protein flexibility, alpha carbon temperature factors are retrieved from the input crystal PDB file or calculated from NMR ensembles (24):

(1) $$\begin{equation*}{B_i} = \frac{{8{\pi ^2}}}{3} \cdot {{\rm RMSF}_i^2},\end{equation*}$$

where RMSF is the root-mean-squared fluctuation obtained from the different models present in the NMR ensemble for a given alpha carbon. Also, Gaussian (GNM) (25) and anisotropic (ANM) (26,27) elastic network models are calculated as implemented in the ProDy stand-alone package (28) to obtain theoretical *B*-factors for each residue. The *B*-factor values of each residue in a protein segment (secondary structure or super-secondary structure elements) are averaged to obtain the flexibility values of such segments. Coordinated motions in the protein are predicted using ANM residue-to-residue cross-correlation values. Such values are used to calculate protein segment cross-correlation values as previously described (11).

### Exploration of the grafted region possible boundaries

The input protein structures are superimposed using combinatorial extension (CE) (29). From such superimposition, a sequence pairing between the two proteins is derived from aligned structures by greedily pairing the closest alpha carbons from each of the input chains (residue doublet). Such atoms have to be within a distance cut-off of 1.9 Å. Residue doublets that do not respect amino acid correlativity in both input proteins are unpaired (and thus gapped). Residue doublets with a difference in sequence position four times higher than the distribution standard deviation are also unpaired.

For each loop region to be grafted (understood as the super-secondary structure formed by the coil segment and its flanking regular secondary structures), all the different residue doublets on the flanking SSEs represent a potential boundary for the grafting experiment. Thus, different lengths of the flanking SSEs might be preserved from the scaffold, but the resulting graft design will contain the whole coil region from the insert protein. A boundary is defined on a given position only if such a position can be matched by the previously described sequence pairing. If such position corresponds to an unpaired residue (be it on the scaffold or on the insert proteins), the new boundary is defined by pairing the unaligned residue with the closest flanking residue from the sequence where the gap is. A particular pair of boundaries (N- and C-terminal) is only accepted for further evaluation if the grafted variant provides any sequential variation on the scaffold protein or any other accepted design.

### Generation and evaluation of 3D structure models

3D models for each of the constructed grafted variants to be evaluated are built using MODELLER (30,31). The inputs of the program are (i) the 3D structures of the proteins (templates) from which optimal spatial restraints are derived, (ii) the sequence of the target ‘problem protein’ (in the present work, the designed grafted sequence) for which a 3D model is to be built and (iii) a sequence alignment between all of them. The sequence alignment is used to indicate both templates that need to satisfy spatial restraints. Assuming the existence of some geometric overlay of the scaffold and insert flanking SSEs around the grafted area (6), the ideal model should incorporate spatial restraints from both template proteins on these overlaid regions.

However, only the insert input protein should inform the grafted coil segment and only the scaffold one the rest of the model. To meet these requirements, the coil parts from the 3D template of the scaffold protein are removed, and the 3D templates for the insert protein correspond to the coil region with flanking secondary structures. To align these insert templates to the target grafted sequence, the 3D coordinates of each insert fragment to be grafted are superimposed again on the scaffold 3D structure using CE (29). In comparison to the whole structure superimposition, here only the fragments to be grafted are considered, and only one fragment at a time. From each of these superimpositions, a sequence paring is derived as described, which is used to derive the sequence alignment required by MODELLER.

MODELLER provides a 3D model of the grafted design and a discrete optimized protein energy (DOPE) (32) value to evaluate the goodness of the model. In order to obtain an independent assessment, each generated model is minimized by Rosetta FastRelax (33) using rigid backbone and default constraints, and Talaris2014 as a scoring function (34). The combined score returned by the program is also used to evaluate the stability of the models.

### Comparison to loop remodelling services

The 3D structure of the loop-grafted luciferase/haloalkane dehalogenase bifunctional enzyme (11) was downloaded from the RCSB PDB (20) (PDB ID 6s97). Residues were renumbered so that the first residue in the structure corresponded to residue number 1, and the coordinates corresponding to the sequences _123_IVHMESVVDVIESWDEWPDIEEDIALIK_154_ and _206_WPREIPIKGDGPEDV_220_ were removed from the structure (note a 11-position shift in comparison to the deposited PDB structure). This structure and the sequence corresponding to the chimeric protein carrying the luciferase sequence for these regions (Supplementary Data) were given as input to the ‘Advanced’ mode of use of DaReUS-Loop (18). The best models produced were collected, superimposed on the crystal structure from PDB ID 6s97 (11) and the all-atom RMSD of the two grafted regions was calculated.

## RESULTS

### Web server usage

#### Structure input

On the web server home page, the user is required to provide the coordinates of two different proteins: the *scaffold* from which the loop(s) will be removed and the *insert* from which such loop(s) will be transplanted. These structures can either be uploaded by the user from their local file system or be retrieved from RCSB PDB (20). Upon loading, the SSEs of the input proteins are automatically calculated using DSSP (21,22), and the two proteins are superimposed using the CE algorithm (29).

**Figure 1.**
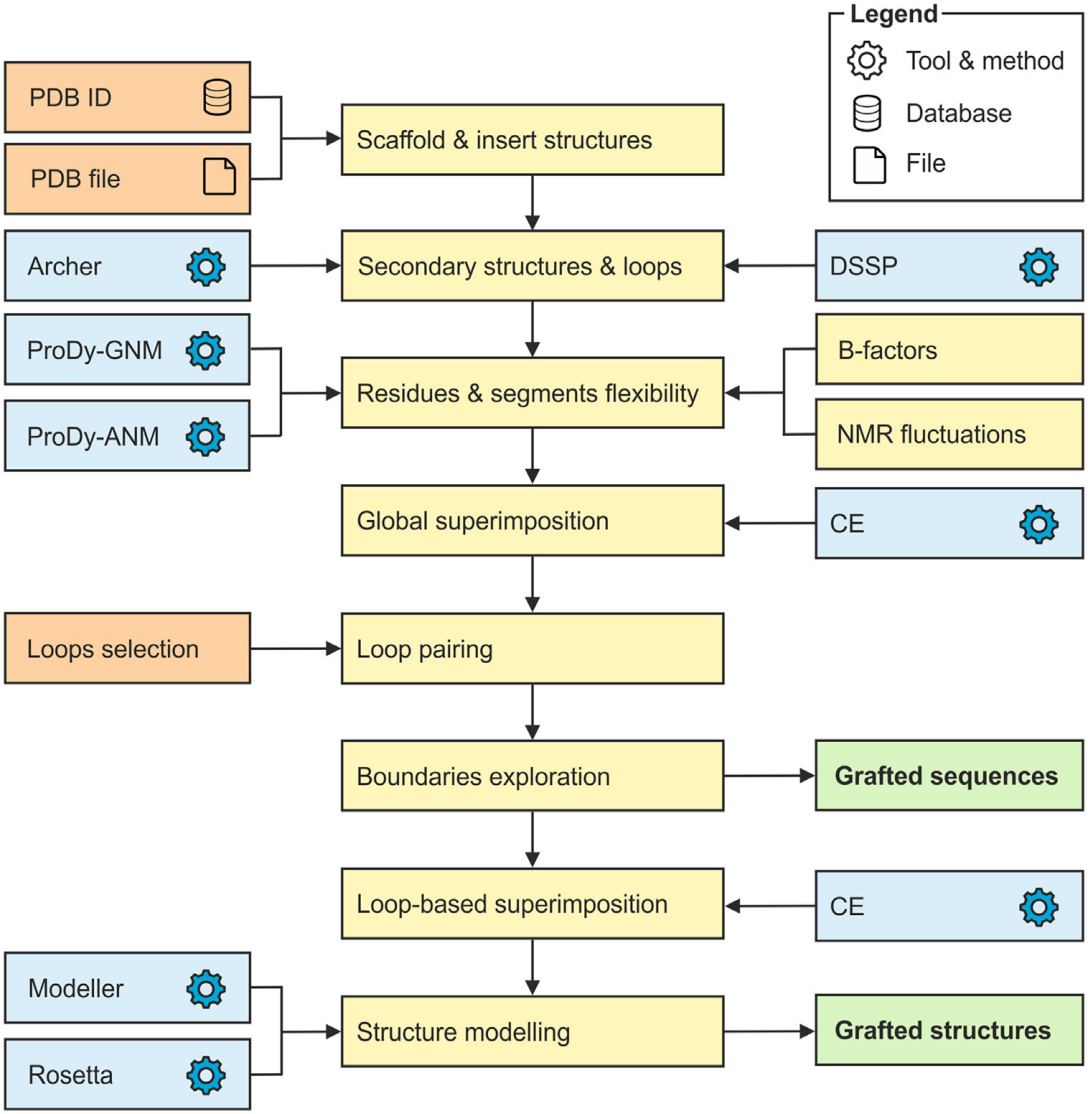
Scheme of LoopGrafter workflow. Scaffold and insert 3D input structures can be fetched from the RCSB PDB or uploaded from a local file system by the user. Secondary structures and loop geometries are calculated on input structures using DSSP and Archer definitions. Flexibility and cross-correlations are calculated by elastic network models using ProDy-GNM and ProDy-ANM. Flexibility analyses (Figure 2) are followed by structural superimposition using CE and pairing of loops in the two input proteins. After systematic exploration of possible grafting boundaries (recombination points), the server provides a list of designed sequences. Finally, a local structural superimposition is used to guide the generation of 3D models for each of the designed sequences, and each of the models is evaluated with MODELLER and Rosetta scores.

LoopGrafter web server is designed to assist the user in the loop grafting effort in six separate steps: (i) secondary structure definition; (ii) loop exploration; (iii) flexibility evaluation; (iv) correlation evaluation; (v) loop pairing; and (vi) loop grafting (Figure 1). The user’s progress through these six steps is permanently monitored in a flow chart presented at the top right corner of the web server page. The completion of this workflow is required to start the loop grafting calculations. The outcome of these calculations is provided in an intuitive visual interface on the Results page.

**Figure 2.**
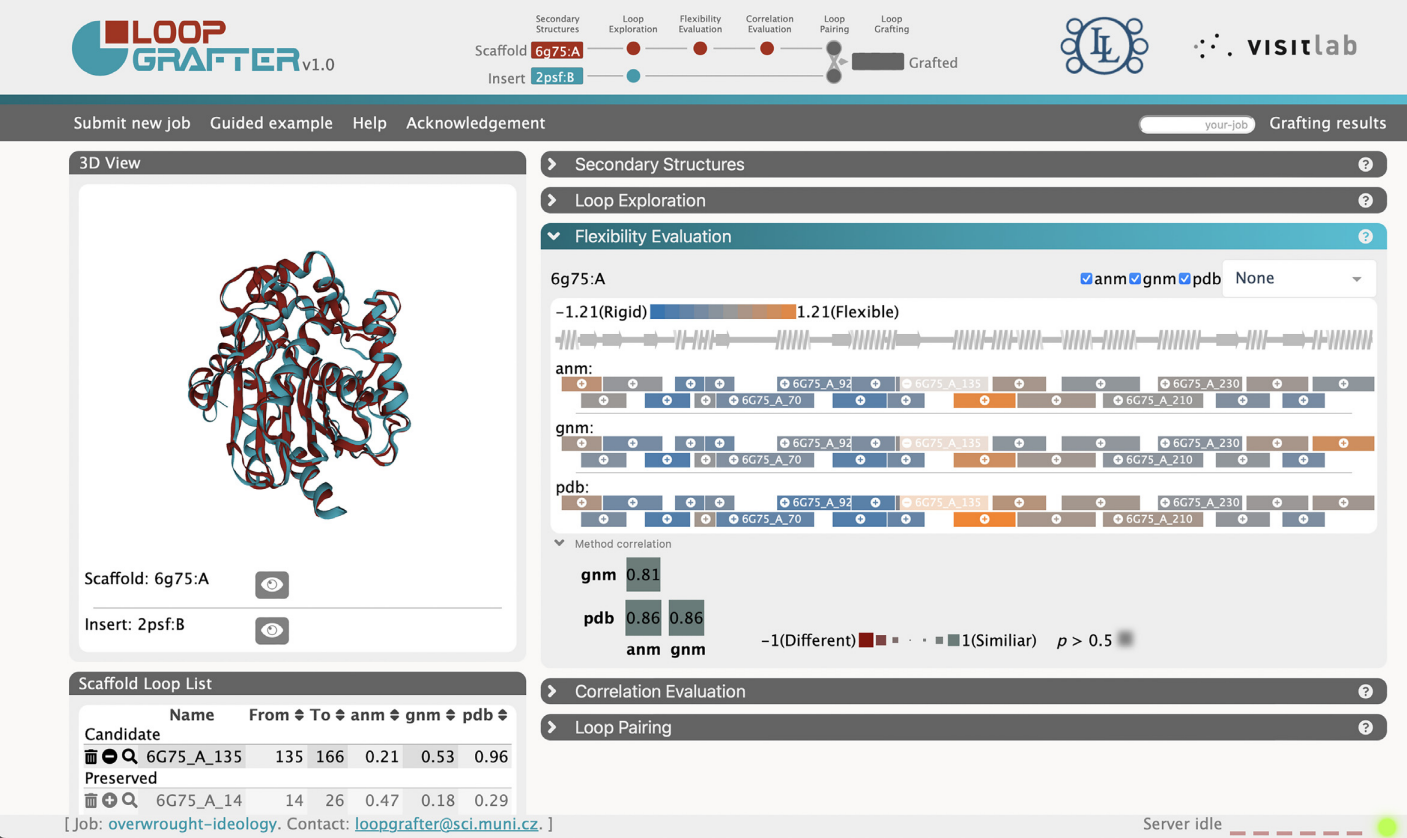
Graphical user interface of LoopGrafter server at the ‘Flexibility evaluation’ step. In the central part of the top banner, a diagram of the grafting pipeline shows the current grafting progress on the workflow. On the 3D view (upper left), the two input proteins are superimposed, and loops can be zoomed for inspection. On the main panel, the *flexibility evaluation* of the scaffold protein is presented. Previously defined loops are represented as rectangles below the 2D representation of the protein, and are coloured according to the *B*-factors either provided by the crystal (pdb) or calculated by elastic network models (anm and gnm). A plus (+) sign on each of the boxes allows for easily including the corresponding loop to the list of loops to be grafted. In the lower part of the panel, a graphical comparison of how the different methods used for assessing flexibility correlate among themselves is provided. In the lower left part, below the 3D view box, the server lists the loops in the scaffold protein indicating which of them are selected for grafting.

- *Secondary structure definition*: As the proteins are uploaded, the secondary structure assignment can be visualized in a two-dimensional (2D) representation presented under the ‘Secondary structure’ caption, and their structures are shown in the 3D viewer situated at the upper left side of the web server page. At this step, the user is allowed to redefine such SSEs, by selecting regions on the 2D representation and changing its secondary structure assignment to *sheet*, *helix* or *coil*.
- *Loop exploration*: Super-secondary structures consisting of two consecutive regular SSEs and the coil segment linking them are automatically extracted from the input proteins. Their geometries are calculated as described in ArchDB (23) upon user’s request (by pressing the ‘Compute’ button). Once calculated, individual loops are represented as square brackets over and below the 2D representation of the input proteins. These brackets can be clicked to select individual loops and display their geometrical details: *distance* (*D*), and *hoist* (*δ*), *packing* (*θ*) and *meridian* (*ρ*) angles, which are the parameters describing the geometry of the loop (23). The selected loops can be zoomed and highlighted in the 3D viewer (upper left in the web server page) by clicking on the box-like 3D view button. Furthermore, when multiple loops are selected, these parameters can easily be compared in the ‘Geometry comparison view’, which presents a bar chart for comparing distances and dial graphs for inspecting the different angles. During this step, the user can define their own loops by clicking and dragging over the 2D view of any of the two input proteins, thus making a new loop selection that can be identified by a user given name. This feature is especially relevant to avoid small structure elements that might be detected in the region(s) of interest for grafting.
- *Flexibility evaluation*: Since a rational driver for loop grafting efforts is to optimize the dynamical behaviour of the grafted region (10–16), LoopGrafter provides the users with a quick assessment of the flexibility of the scaffold protein based on experimental data and elastic network models (28). The user can select which of these flexibility assessment sources he or she is interested in, and the flexibility values are automatically calculated for every alpha carbon in the scaffold protein. In the case of input NMR ensembles, *B*-factors are calculated from the root-mean-squared fluctuations comprised in the ensemble. For each selected flexibility assessment source method, flexibility values can be displayed in the 2D representation of the protein by selecting the preferred representation from the combo box at the top right corner of the ‘Flexibility evaluation’ section. Flexibility values are colour coded according to a colour-scale scheme shown in the upper right, and can be displayed for each alpha carbon in the scaffold protein or averaged per each SSE. Below, the averaged values per super-secondary structure (loop) are shown in separated boxes according to the same scale. Each of such boxes contains a plus (+) sign that enables the selection of that particular loop as a candidate to be grafted. Any loop can be selected for grafting regardless of their assessed flexibility. Below the 3D viewer, the candidate loops to be grafted and the preserved ones are listed during the whole process. Finally, at the bottom of this section, the agreement between the different flexibility methods is presented in the form of correlation values (Figure 2).
- *Correlation evaluation*: Once one or more loops are selected as candidates to be grafted, their correlated movements with other parts of the protein can be assessed in this section of the workflow. Here, two different values are presented: (i) the cross-correlation of the selected loops (super-secondary structures) to any other loops in the protein, shown in light shade, and (ii) the cross-correlation of the coil segment of the selected loops to any SSE in the other loops of the protein. The first metric gives an idea of the overall accord of the motion of the selected loops with others. Except in the case of loops present in regions with an important dynamic gradient (12), these cross-correlations are typically positive and large for neighbouring loops. In contrast, the second metric focuses on the coil segment of the selected loops, which is usually more flexible and the main target of engineering. This segment-centred metric gives a more precise idea of the possible reciprocal conformational influence between the selected loop and other regions in the protein. The cross-correlation values are colour coded and range from −1 (red hue, perfect anti-cross-correlation) to 1 (blue hue, perfect cross-correlation). If by means of this exploration, a strong cross-correlation between the selected loops and other loops in the scaffold protein is detected, the inclusion of such cross-correlated loops as candidates to be grafted might be considered.
- *Loop pairing*: Each of the selected loops to be grafted in the scaffold protein is paired to an equivalent loop in the insert protein during this step, which can be done either automatically or manually. The user is presented with the 2D representations of the two proteins enriched with a display of the selected loops for grafting in the scaffold protein and all the loops in the insert protein. If the manual pairing is chosen, the user is required to pick a replacement equivalent from the insert protein for each of the selected loops. Upon selection of such replacement or after automatic pairing, a comparison of the geometrical parameters of the scaffold and insert loop is presented (as described earlier in the ‘Loop exploration’ step). It has to be noted that the paired loops need to be structurally equivalent. This means that the pair of loops needs to be reasonably overlaid in space after the CE superimposition (28) of the two input proteins (see the ‘Exploration of the grafted region possible boundaries’ section). When the selected loops are not structurally compatible, the server will not prevent the user from proceeding to the next step, but the number of presented solutions will be dramatically reduced or, more likely, will be none (see below). This task can easily be guided by the 3D superimposition of the input proteins available in the 3D viewer.
- *Loop grafting*: The last step of the workflow consists of preparing the required information for producing 3D models of the different possibilities for grafting the selected loops and assessing their stability. First, after pressing the ‘Compute grafting sequences’ button, all the possible boundaries in the grafted region(s) that generate different sequences are explored (see the ‘Materials and Methods’ section). After this initial exploration, the user is informed about the total number of different grafting variants the server will generate and their sequences can be downloaded in JSON format. Compared to available loop remodelling tools (17–19), LoopGrafter does not require the input of the sequence of the final chimeric protein design. Instead, it explores all possibilities for recombination points around the region(s) where the insert structure needs to be placed. Then, after acknowledging ownership of licenses for MODELLER (30,31) and optionally for Rosetta (33), the calculations can be started by clicking on the ‘Start modelling’ button. It has to be noted that MODELLER is required to generate 3D models for each of the grafted variants and to calculate DOPE scores (32), and that Rosetta FastRelax is required to obtain Rosetta scores (34). Both tools are free for academic users. A bookmarkable link is provided to track the progress of the calculations. It can also be retrieved at any moment by introducing the session ID (permanently shown in the lower left corner of the LoopGrafter web interface) in the ‘Grafting results’ box present in the right part of the upper horizontal menu under the grafting workflow scheme.

#### Results

Following the link provided in the previous step, the user is directed to a query-tracking progress bar that informs about the current state of their query. When all the calculations are done, the Results page is accessible through the query-tracking interface or by inputting the session identifier (displayed at the bottom left corner of the web interface) in the ‘Grafting results’ box on the upper horizontal menu. The Results page presents an overview of all calculated grafting variants (‘Experiments’), their DOPE and Rosetta FastRelax scores, the number of inserts in the resulting grafting variant and its number of amino acids (length). The scores and the number of inserts can be used to sort the obtained variants, and any individual variant can be selected for 2D and 3D visualization, showing the scaffold and insert regions colour coded. Results can be downloaded in three different forms: (i) the 3D structure coordinates of any selected variant (‘Download solution PDB’; (ii) a digest of all variants scores and sequences (‘Download score table’); and (iii) the compressed bundle of all results, including the 3D structure models generated by MODELLER and Rosetta for each grafting variant (‘Download all solutions’).

### Use case: transplanting two loops from *Renilla* luciferase to a bifunctional ancestor of luciferase and dehalogenases

The grafting strategy presented here has been successfully implemented in grafting a particular loop from the bioluminescent *Renilla* luciferase (35) to a structurally related scaffold. The scaffold was a bifunctional reconstructed ancestral protein catalysing both a hydrolytic dehalogenation of small halogenated compound and a light-emitting monooxygenation of a bulky coelenterazine molecule (36). Based on the previous observation of the dynamic behaviour of one particular loop in a dynamic hinge region (a loop connecting the rigid main domain with the flexible cap domain of the protein), the transplantation of such loop resulted in a grafted engineered protein (PDB ID 6S97) with improved bioluminescence. In more detail, scaffold residues 146–166 were replaced with residues 148–167 from the insert, and the grafting variant rendered a 40 000-fold more efficient bioluminescent reaction when compared to that of the scaffold enzyme. Furthermore, the grafting variant had a higher affinity towards the substrate and lower product inhibition compared to *Renilla* luciferase, but only reached 20% of its efficiency (11).

#### Data input

The grafting approach reported there can be replicated using the LoopGrafter. First, in the ‘Protein upload’ step the bifunctional ancestor should be introduced as scaffold and *Renilla* luciferase as insert. The asymmetric unit of the bifunctional ancestor (PDB ID 6G75) consists of two relatively similar conformers, so any of the chains can be inputted to the server (here, chain A will be considered). In contrast, the asymmetric unit of *Renilla* luciferase (PDB ID 2PSF) contains two different conformers, one corresponding to an open cap domain (chain A) and the other corresponding to a closed one (chain B). Since the closed cap domain (chain B) is more similar to the scaffold bifunctional ancestor, it should be chosen as input for the insert protein. The global similarity between the proteins should be noted: scaffold and insert only differ by 1.27 Å RMSD after CE superimposition and 2% in sequence length. Crucially, the similarity values recommended for using LoopGrafter are <8 Å RMSD and <20% difference in sequence length.

The two input proteins result in different secondary structure assignments in the N-terminal region connecting the main and cap domains (residues 135–167 in the scaffold protein). Particularly, a small helical element is present in the scaffold (residues 151–153) but not in the insert protein. Since this region is important for the grafting experiment, the helical structure can be deleted from the scaffold protein in this step. Alternatively, a user-defined loop bypassing the small helical structure can be defined on the scaffold protein in the next step. In case such knowledge about the importance of the region is not available at this moment of the process, the steps in the web server can always be traced back and one of the two strategies can be used. In this example, the region 151–153 from the scaffold protein was selected and set to ‘coil’.

#### Flexibility assessment

After calculating loops and their geometries in the second step (‘Loop exploration’ step), the flexibility and dynamic properties of the scaffold protein can be assessed in the third one (‘Flexibility evaluation’ step). From this analysis, the loop 6g75_A_135 can be identified as a key element connecting stationary and dynamic parts of the scaffold protein. This loop corresponds to loop 9 in (11) and is the loop the authors successfully grafted to confer the scaffold protein a more efficient bioluminescence. Also, it is noteworthy that this loop plays a key role in opening and closing the active site pocket of both input proteins (11). This loop can be marked for inclusion in the grafting protocol by clicking on the plus symbol on it. It is worth noting that, regardless of the flexibility values calculated, any loop can be included in the grafting protocol. Thus, loops bridging more rigid or more flexible parts of the input protein could be grafted as well.

Other regions in the scaffold protein that have coordinated movements with the selected loop(s) can be identified in the ‘Correlation evaluation’ step. In this example, the loop 6g75_A_210 strikingly has a notable cross-correlation with the motions of the coil segment of the previously selected loop (*R* = 0.42). This loop was not considered in the original grafting effort on the bifunctional ancestral scaffold. However, because of being involved in the joint dynamic system of opening and closing of the active site pocket, its transplantation brought the bioluminescence efficiency levels of the scaffold to the level of optimized commercially available luciferase (Toul *et al.*, in preparation).

#### Grafting preparation

The final steps on the server before the actual grafting involve pairing the loops to be grafted from the scaffold protein with their equivalents in the insert protein. In this example, the equivalent insert loops to 6g75_A_135 and 6g75_A_210 are 2psf_B_137 and 2psf_B_211, respectively, which can be paired manually or automatically. The steps described here lead to 474 different grafting possibilities, considering the combinations of flanking SSE insertion points for each grafted loop that generate different sequences and all the possible combinations of grafted loops.

#### Results’ evaluation

The 474 chimeric sequences generate their corresponding 3D model scores ranging between 1716 and 2458 units according to MODELLER metric (32) and between −262 and 67 Rosetta energy units (34). These scores are reported in arbitrary units and can be taken as a proxy for the energy of the model, and as a rule of thumb they are better as they get lower. The chimera constructed in the aforementioned publication (11) scored 1839 units in MODELLER and −207 energy units in Rosetta, ranking 4th and 1st out of 18 possible single loop 6g75_A_135 designs. The experimentally attempted design for the second loop, 6g75_A_210, scored 1771 units in MODELLER and −150 energy units in Rosetta, ranking 2nd and 21st out of 24 possible single loop designs, respectively. The combined loop design achieved the score of 1928 units in MODELLER and −197 energy units in Rosetta, ranking 10th and 59th out of 432 possible designs. Overall, these numbers illustrate the capacity of LoopGrafter in spotting successful chimeric graft designs. Another four use cases reproducing previously published experimental data (37–39) are provided in the Supplementary Data.

### Comparison to available loop remodelling tools

In order to illustrate the utility of LoopGrafter, we attempted to reproduce the same case study presented here using DaReUS-Loop (18), a web server for loop remodelling that in its advanced mode of use allows for the generation of chimeric models. Given a gapped 3D structure, and the complete sequence of the protein to model (see the ‘Materials and Methods’ section), it performs a homology search to find structures to cover the gaps in the input 3D structure and generates 3D models for the identified loops, independently for each loop. DaReUS-Loop identifies the *Renilla* luciferase as source for the first loop (6g75_A_135), but not for the second one (6g75_A_210) (Supplementary Table S1). The solution by LoopGrafter (combined loop design) achieved similar or better RMSD than the two solutions by DaReUS-Loop that identified the *Renilla* luciferase as source in the first loop (Supplementary Table S1) when compared to the chimeric crystal structure reported in the case study presented earlier (PDB ID 6s97) (11). Even when the second loop was not grafted in the original publication (11), LoopGrafter results on this loop resemble more the original structure than DaReUS-Loop ones (Supplementary Table S1). Supplementary Figure S1 summarizes this comparison. LoopGrafter and DaReUS-Loop (as a representative of a loop remodelling tool repurposed for loop grafting) greatly differ in input requirements and outputs. Supplementary Table S2 presents a summary of such differences. Among them, it is worth noting that LoopGrafter does not require the chimeric solution (sequence of the resulting protein) as input, but offers all possibilities of chimeras exploring different recombination points along the flanking regular secondary structures of the loops to be grafted.

## DISCUSSION AND CONCLUSIONS

LoopGrafter is a web-based application designed to provide guidance in the process of transplanting loops between homologous, structurally related proteins. The power of this technique in protein engineering has been shown in the past (5,11). Surprisingly, loop-based rational protein engineering lacks systematic support (4,40). Other available tools and approaches addressing the problem of remodelling existing or missing loops in a given protein structure have been recently reviewed (41). However, transplanting a loop between two related proteins represents an entirely different scenario to that of loop remodelling, and thus those tools can rarely help in this particular task.

With the aim of assisting the non-expert in the mission of transplanting loops between structurally related proteins, the server provides a step-by-step procedure at the end of which the user will obtain a number of chimeric designs and structures. The approach successively involves calculating loops and their geometries in the two input proteins, assessing their similarities and dynamics using elastic network models, exploring correlated motions among loops and finally selecting a number of loops to be transplanted. The exploration of the loop dynamics and especially their cross-correlations is often key to explain the function of proteins (15). The way LoopGrafter presents the cross-correlation analysis to the user enables the easy discovery of motion-entangled loops. Indeed, loops lining the access pathways to the enzyme catalytic centre can exert control over different aspects of the kinetics of the enzyme activity (11).

When designing chimeric proteins, one of the most difficult questions is deciding the exact sequence points where to perform the excision. ProtLego is a recent tool designed to generate interdomain chimeras (42) that is exploring all possible excision points where the two related proteins structurally overlap in their single recombination point problem. In comparison, transplanting loops between structurally related proteins requires considering two recombination points for each loop to be transplanted. Even when LoopGrafter has looser requirements for defining such overlapping recombination points (1.9 Å compared to 1 Å in ProtLego), it still considers all possibilities that generate sequence diversity in the pool of chimeric designs. This provides the researcher with a systematic overview of all the possible grafting designs that can be tailored with their query proteins.

Finally, we have showcased that the scoring systems from MODELLER and Rosetta help identifying loop-grafted chimeric designs that encompass structural and biological meaning. Among all generated chimeric sequences, based on the provided scores, the user can select the designs more likely to be successful in the lab. In the use case described here, experimentally successful loop-grafted chimeric proteins were favourably scored by both methods (11). The tool has been thoroughly validated against four additional biomolecular systems with grafted loops (Supplementary Data): monomeric triosephosphate isomerase (37), immunoglobulin-like β-sandwich protein tenascin (38), tyrosine phosphatases and *Streptococcus pyogenes* sortase A (39). In these four instances, LoopGrafter was able to identify the exact excision sequence (or one that is very close to) that was used in the experimental loop transplantation exercises. Other domains in which we envisage LoopGrafter could be exploited are CDR transplantation in antibody design (i.e. for antibody humanization) and the transplantation of loops with the purpose of designing inhibited (non-catalytic) protein variants and to tailor protein inhibitors for proteases. Relying on these strengths, we are positive that LoopGrafter will be a useful tool in helping the experimental efforts of protein and metabolic engineers. Future developments can focus on loop grafting in heterologous protein structures exploiting geometrical search engines (8,43) to fetch compatible loops from the PDB (20) and improving scoring functions for more reliable identification of functional designs.

## DATA AVAILABILITY

LoopGrafter is a web server available at https://loschmidt.chemi.muni.cz/loopgrafter.

## Supplementary Material

gkac249_Supplemental_File
