# Supplementary material for: LoopGrafter: a web tool for transplanting dynamical loops for protein engineering
Source: Nucleic Acids Res. 2022 Apr 19;50(W1):W465–73. doi: 10.1093/nar/gkac249 (PMC9252738; doi:10.1093/nar/gkac249)
Supplement: gkac249_Supplemental_File [file gkac249_supplemental_file.pdf]

LoopGrafter: A Web Tool for Transplanting Dynamical Loops for Protein Engineering

Joan Planas-Iglesias[1,2§], Filip Opaleny[3§], Pavol Ulbrich[3], Jan Stourac[1,2], Zainab Sanusi[1], Gaspar P. Pinto[1,2], Andrea Schenkmayerova[1,2], Jan Byska[3], Jiri Damborsky[1,2], Barbora Kozlikova[3*] and David Bednar[1,2,*]

[1] Loschmidt Laboratories, Department of Experimental Biology and RECETOX, Faculty of Science, Masaryk University, Brno, Czech Republic

[2] International Clinical Research Center, St. Anne's University Hospital Brno, Brno, Czech Republic

[3] Department of Visual Computing, Faculty of Informatics, Masaryk University, Brno, Czech Republic * To whom correspondence should be addressed. Tel: +420605143394; Fax: +420549496302; Email: 222755@mail.muni.cz

[§]The authors wish it to be known that, in their opinion, the first two authors should be regarded as Joint First Authors.

Supplementary Materials

SEQUENCE OF CHIMERIC HALOALKANE DEHALOGENASE BIFUNCTIONAL ANCESTOR CARRYING TWO GRAFTED LUCIFERASE LOOPS:

ATGDEWWAKCKQVDVLDSEMSYYDSDPGKHKNTVIFLHGNPTSSYLWRNVIPHVEPLARCLAPDLIG
MGKSGKLPNHSYRFVDHYRYLSAWFDSVNLPEKVTIVCHDWGSGLGFHWCNEHRDRVKGIVHMES
VVDVIESWDEWPDIEEDIALIKSEAGEEMVLKKNFFIERLLPSSIMRKLSEEEMDAYREPFVEPGESRR
PTLTWPREIPLVKGGKPDVIEIVKSYNKWLSTSKDIPKLFINADPGFFSNAIKKVTKNWPNQKTVTVKG
LHFLQEDSPEEIGEAIADFLNELT

SUPPLEMENTARY TABLES AND FIGURES

Table S1. Comparison of LoopGrafter and DaReUs Loop results on the case study

Web Server	Loop Grafted	Loop Source (PDB ID chain)	Source protein	RMSD (Å) (atoms compared)
LoopGrafter	6g75_A_135	2PSF B	Luciferase	1.874 (232 atoms)
DaReUs Loop	6g75_A_135	2PSE A	Luciferase	1.834 (227 atoms)
DaReUs Loop	6g75_A_135	2PSF A	Luciferase	4.064 (227 atoms)
LoopGrafter	6g75_A_210	2PSF B	Luciferase	1.064 (108 atoms)
DaReUs Loop	6g75_A_210	2WAF A	penicillin-binding protein 2B	4.049 (64 atoms)
DaReUs Loop	6g75_A_210	1PIY A	ribonucleotide reductase R2	4.495 (64 atoms)

Table S2. Comparision of LoopGrafter and DaReUs Loop inputs and outputs

Feature	LoopGrafter	DaReUsLoop
**Input scaffold protein**	Complete PDB structure	Gapped PDB structure
**Input insert protein**	Complete PDB structure	Chimeric
**Combined grafted loop output structure**	Yes	No
**Output structures type**	Single source, multiple recombination points	Multiple sources, single recombination point

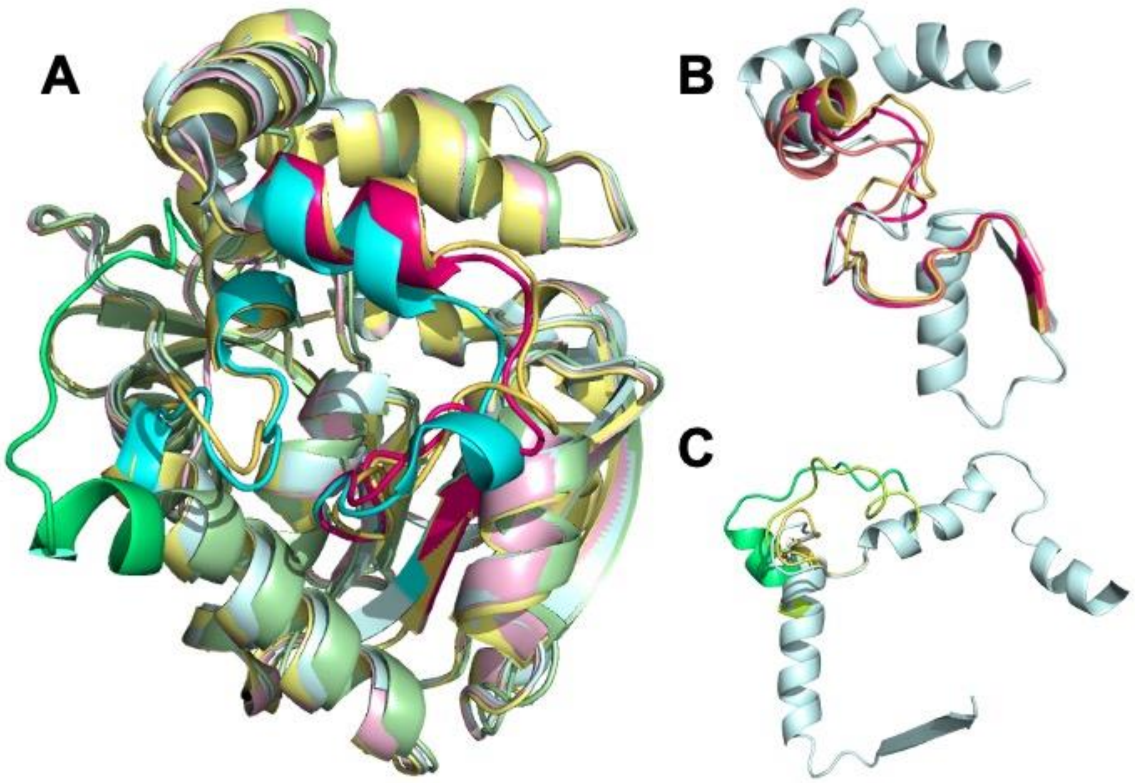

Figure S1. Comparison of LoopGrafter and DaReUs Loop. Structural superimposition of selected results from DaReUs Loop (red and green hues) and the combined LoopGrafter presented in the use case (yellow) to the crystal structure of the loop 6g75_A_135 chimeric bifunctional luciferase/haloalkane dehalogenase protein (cyan) reported in (12) (PDB ID 6s97). **A**. Best DaReUs Loop solutions for loop 6g75_A_135 (magenta) and for loop 6g75_A_210 (green). Grafted regions are shown in bright hues, the rest of the protein in paler ones. **B**. Detail of loop 6g75_A_135. The two best solutions from DaReUs Loop are shown in red hues. **C.** Detail of loop 6g75_A_210. The two best solutions from DaReUs Loop are shown in green hues.

ADDITIONAL USE CASES:

We used LoopGrafter to attempt to reproduce loop transplantation results reported in the literature with different degrees of success. One of the major problems we faced is that usually experimental efforts are focussed on transferring the coiled-coiled segments of the loop super-secondary structures.On the other hand, LoopGrafter requires reference points in the flanking regular secondary structures to create recombination points. In order to increase the chances to replicate the experimental results, the definition of secondary structures can be tuned (extended towards the coiled-coil region) to allow for recombination points inside the coiled-coil region. This strategy has been used in some of the use cases presented below.

Stabilizing a catalytic loop in monomeric triosephosphate isomerase (mono-TIM):

Loop 1 in triosephosphate isomerase contains one of the catalytic residues of the enzyme (K13). However, the loop of the monomeric form of the enzyme is too flexible, hampering its catalytic properties when compared to the activity levels achieved by its dimeric form. The catalytic loop was truncated by one-residue to rigidify the loop, matching some of the desired structural features but unfortunately not increasing the activity of the monomeric form of the enzyme (1).

Here, we will task LoopGrafter to replace the target loop from the monomeric triosephosphate isomerase with the resulting shorter loop in the engineered protein. It has to be noted that the crystal structures described in the aforementioned reference study (PDB ID 1TTJ and PDB ID 1MTM) (1) have missing residues, thus we have used homologous structures with complete sequences (PDB ID 2WSR and PDB ID 4PC8, respectively)

Input proteins:

Scaffold: PDB ID 2WSR, chain A. Excised sequence: $_{14}$CNGSQQSL$_{21}$

Insert: PDB ID 4PC8, chain A. Inserted sequence: $_{14}$SGSPDSL$_{20}$

Secondary structure adjustment: None

The grafting attempt generates only one solution, which corresponds exactly with the sequence attempted by the original authors. While the sequence obtained is 7 residues longer at the N-terminal end (IAAANWK) and 9 residues longer at the C-terminal one (LSELIDLFN), these residues are conserved across the two input proteins and, thus, their inclusion in the grafted segment is irrelevant. The solution scored 1458 units in MODELLER and -247 units in Rosetta, which are low scores indicative of a likely successful grafting variant.

Rosetta loop re-design in the immunoglobulin-like β-sandwich protein tenascin:

Residues 22-31 from the fibronectin type III domain in tenascin-C were exchanged by three structurally compatible loops (A, B, and C) using a Rosetta-based protocol designed by the authors (2). Loop B in particular produced a chimeric protein with similar stability to the wild type for which the Authors obtained a crystallographic structure. The source structure from the grafted loops is not stated in the original work, and thus we task LoopGrafter with transplanting into the tenascin scaffold (PDB ID 1TEN) from loop B crystal (PDB ID 2RB8).

Input proteins:

Scaffold: PDB ID 1TEN, chain A. Excised sequence: $_{821}$FKPLAEIDGI$_{832}$

Insert: PDB ID 2RB8, chain A. Inserted sequence: $_{821}$MPPSQPVDGF$_{832}$

Secondary structure adjustment:

Scaffold: Both flanking regular β-strands extended: residues 824-827: sheet; residues 829-832: sheet.

Insert: Both flanking regular β-strands extended: residues 824-827: sheet; residues 829-832: sheet.

The grafting attempt generates five different solutions, one of which corresponds exactly with the sequence attempted by the original authors. The best scoring one (MODELLER score 558 units , Rosetta score -4 units), corresponds to a sequence two residues shorter than the one attempted in the original publication, where the extreme positions of the insert M821 and F832 are deleted. The second one (scoring 781 units in MODELLER and 111 units in Rosetta) corresponds to the insert sequence with the position F832 deleted. The two grafted sequences are four residues longer in the N-terminal end, but those 4 residues are common in the insert and the scaffold, making the results close matches with the sequence attempted in the original publication (2). It has to be noted that the scores we obtained for Rosetta significantly differ from the ones obtained in the original publication (score -149 units). However, the scores yielded by MODELLER are extremely low, indicating that the chimeras are likely to be successful.

Grafting WDP loop in protein tyrosine phosphatases

Members of the protein tyrosine phosphatase type II family possess a well-conserved catalytic loop (WPD loop) characterized by its particular flexibility and triad of conserved amino acids, Trp-Pro-Glu, the third of which acts as an acid/base catalyst. Previous studies focused on the human and *Yersinia pestis* members of the family suggested that the conformational dynamics of the WPD loop is key to describe the catalytic capacities of the enzyme (3). To further understand the role of the non-conserved positions in the WPD loop, the Authors undertook a wide exploration of the possible recombination points to transplant a catalytic loop from *Yersinia pestis* protein tyrosine phosphatase to its human homolog. Few of the attempted chimeras were soluble, and their characterisation revealed different levels of activity(4).Among the multiple findings in this study, the authors point at the role of E186 in the control of the overall flexibility of the loop.

Input proteins

Scaffold: PDB ID 3I80, chain A. Excised sequence: $_{175}$HYYWPDFGVPE$_{186}$

Insert: PDB ID 1YPT, chain A. Inserted sequence: $_{346}$HVGNWPDQTAVS$_{357}$

Secondary structure adjustment:

Scaffold: Both flanking regular secondary structures extended: residues 176-180: sheet; residues 185-188: helix.

Insert: Both flanking regular secondary structures extended: residues 350-355: sheet; residues 360-362: helix.

The grafting attempt generates 65 different solutions, none of them corresponding exactly to the chimeras the Authors attempted in the aforementioned study (4). Unfortunately, the grafting efforts of the study were focused in a too narrow region for LoopGrafter to be able to reproduce the exact same results. However, it is worth noting that the shortest chimeras we obtained on the C-terminal end included E186 from the human scaffold protein, a key residue to explain the dynamics of the loop. Particularly, the substitution of the fragment $_{75}$HYYWPDFGVP$_{185}$ in the human scaffold by the fragment $_{346}$HVGNWPDQTAV$_{356}$ from the *Yersinia* insert contains the key residue E186 and is only 2 residues different from a soluble and active chimera, obtained the first ranking solution by MODELLER (score 1148 units) and the second ranking one by Rosetta (score -308 units).

Engineering the specificity of *Streptococcus pyogenes* sortase A by loop grafting

Sortases are enzymes present in the cell wall of Gram-positive bacteria responsible for the attachment of virulence factors upon the recognition of pentapeptide motives in the target peptidoglycan. The authors of the study queried whether the promiscuous behaviour of sortase A from *Streptococcus pyogenes* could be transferred to *Staphylococcus aureus* by transplanting the loop β7/β8 (5). In order

to design the recombination points, the authors relied on a structure-based alignment using the constraint-based multiple alignment program COBALT (6).

Input proteins
Scaffold: PDB ID 3FN5, chain A. Excised sequence: $_{211}$IEATE$_{215}$
Insert: PDB ID 2KID, chain A. Inserted sequence: $_{187}$YNEKTGVWEK$_{196}$

Secondary structure adjustment:
Scaffold: N-terminal β-strand extended: residues 208-211: sheet.
Insert: Both flanking regular β-strands extended: residues 184-190: sheet; residues 195-197: sheet.

The grafting attempt generates 19 different solutions, and none of them corresponds to the one that the authors attempted in their study (5). Here, it has to be noted that the alignment produced by COBALT (6) and the alignment used by LoopGrafter are strikingly different and thus obtaining the exact same solution is impossible. Particularly, the alignment differs in the C-terminal region of the loop, wherein there is a shift of two positions of the aligned residues in the scaffold protein. However, the best ranking solution both in MODELLER (score 1211 units) and Rosetta (score -70 units) corresponds to the excision of $_{211}$IEATERII$_{218}$ from the *S. aureus* scaffold and the subsequent insertion of the sequence $_{187}$YNEKTGVWEKR$_{196}$ from *S. pyogenes*. This solution is structurally very close to the one attempted by the Authors of the original study. This example illustrates LoopGrafter capabilities for proposing grafting chimeras with significantly different sequence length than the input scaffold protein.

References

1. Thanki, N., Zeelen, J.P., Mathieu, M., Jaenicke, R., Abagyan, R.A., Wierenga, R.K. and Schliebs, W. (1997) Protein engineering with monomeric triosephosphate isomerase (monoTIM): the modelling and structure verification of a seven-residue loop. *Protein Engineering, Design and Selection*, **10**, 159-167.
2. Hu, X., Wang, H., Ke, H. and Kuhlman, B. (2007) High-resolution design of a protein loop. *Proceedings of the National Academy of Sciences*, **104**, 17668-17673.
3. Moise, G., Morales, Y., Beaumont, V., Caradonna, T., Loria, J.P., Johnson, S.J. and Hengge, A.C. (2018) A YopH PTP1B Chimera Shows the Importance of the WPD-Loop Sequence to the Activity, Structure, and Dynamics of Protein Tyrosine Phosphatases. *Biochemistry*, **57**, 5315-5326.
4. Ruidan Shen, R.M.C., Keith J. Olsen, Teisha Richan, Tiago A. S. Brandão, Ryan D. Berry, Alex Tolman, J. Patrick Loria, Sean J. Johnson, Shina Caroline Lynn Kamerlin, Alvan C. Hengge. (2021) Insights into the Importance of WPD-Loop Sequence for Activity and Structure in Protein Tyrosine Phosphatases. *ChemRxiv*.
5. Wójcik, M., Szala, K., van Merkerk, R., Quax, W.J. and Boersma, Y.L. (2020) Engineering the specificity of Streptococcus pyogenes sortase A by loop grafting. *Proteins: Structure, Function, and Bioinformatics*, **88**, 1394-1400.
6. Papadopoulos, J.S. and Agarwala, R. (2007) COBALT: constraint-based alignment tool for multiple protein sequences. *Bioinformatics*, **23**, 1073-1079.
7. P Karami, Y., Rey, J., Postic, G., Murail, S., Tufféry, P. and de Vries, S.J. (2019) DaReUS-Loop: a web server to model multiple loops in homology models. *Nucleic Acids Research*, **47**, W423-W428
8 Schenkmayerova, A., Pinto, G.P., Toul, M., Marek, M., Hernychova, L., Planas-Iglesias, J., Daniel Liskova, V., Pluskal, D., Vasina, M., Emond, S. *et al.* (2021) Engineering the protein dynamics of an ancestral luciferase. *Nature Communications*, **12**, 3616.
